# Colorectal Cancers Mimic Structural Organization of Normal Colonic Crypts

**DOI:** 10.1371/journal.pone.0104284

**Published:** 2014-08-11

**Authors:** Laura Cernat, Cristina Blaj, Rene Jackstadt, Lydia Brandl, Jutta Engel, Heiko Hermeking, Andreas Jung, Thomas Kirchner, David Horst

**Affiliations:** 1 Pathologisches Institut, Ludwig-Maximilians-Universität, München, Germany; 2 Victor Babes University of Medicine and Pharmacy, Timisoara, Romania; 3 Experimentelle und molekulare Pathologie, Pathologisches Institut, Ludwig-Maximilians-Universität, München, Germany; 4 Tumorregister München (TRM), Klinikum der Universität, München, Germany; 5 Institut für medizinische Informationsverarbeitung, Biometrie und Epidemiologie, Ludwig-Maximilians-Universität, München, Germany; 6 Deutsches Konsortium für translationale Krebsforschung (DKTK), Heidelberg, Germany; 7 Deutsches Krebsforschungszentrum (DKFZ), Heidelberg, Germany; University of Kentucky, United States of America

## Abstract

Colonic crypts are stereotypical structures with distinct stem cell, proliferating, and differentiating compartments. Colorectal cancers derive from colonic crypt epithelia but, in contrast, form morphologically disarrayed glands. In this study, we investigated to which extent colorectal cancers phenocopy colonic crypt architecture and thus preserve structural organization of the normal intestinal epithelium. A subset of colon cancers showed crypt-like compartments with high WNT activity and nuclear β-Catenin at the leading tumor edge, adjacent proliferation, and enhanced Cytokeratin 20 expression in most differentiated tumor epithelia of the tumor center. This architecture strongly depended on growth conditions, and was fully reproducible in mouse xenografts of cultured and primary colon cancer cells. Full crypt-like organization was associated with low tumor grade and was an independent prognostic marker of better survival in a collection of 221 colorectal cancers. Our findings suggest that full activation of preserved intestinal morphogenetic programs in colon cancer requires *in vivo* growth environments. Furthermore, crypt-like architecture was linked with less aggressive tumor biology, and may be useful to improve current colon cancer grading schemes.

## Introduction

Colorectal cancer (CRC) derives from normal colonic mucosa by a well characterized, stepwise accumulation of mutations that transform normal epithelial cells into tumor cells with malignant behavior [1]. Normal colonic mucosa is quite simply organized, basically as a sheet of epithelial cells with infoldings forming stereotypical crypts [2]. Each crypt contains distinct functional compartments with stem cells at the crypt base, responsible for continuous renewal of aged epithelial cells, a proliferating transit amplifying zone extending to the mid crypt, and most mature epithelial cells towards the crypt apex [3]. Experimental data, mainly derived from murine small intestinal crypts, suggest high canonical WNT signaling activity within the stem cell compartment at the crypt base [4], [5], while mature cells at the crypt apex are characterized by expression of differentiation markers, such as Cytokeratin 20 (CK20) [6].

In contrast to normal colonic mucosa, the structural organization of CRC is much less well understood. CRCs form tumor masses rather than sheets of cells, with varying degrees of morphologically disarrayed epithelial glands [7]. However, these tumors do not appear to be completely unorganized. A gradient between less differentiated tumor cells at the leading tumor edge, and more glandular differentiated cells in the tumor center can be observed in most CRCs [8], [9]. In addition, tumor cells at the leading tumor edge typically express strong nuclear β-Catenin, indicating high WNT signaling activity, and have been disputed to represent a progenitor cell population of operationally defined colon cancer stem cells [10], [11]. On the contrary, expression of CK20 was suggested to label more differentiated colon cancer cells, since its expression was mutually exclusive with putative colon cancer stem cell markers [11], [12]. Collectively, these data suggest some degree of structural organization within CRC, with cancer stem cells at the leading tumor edge and differentiation towards the tumor center.

Considering these findings and known compartments within intestinal crypts, we herein investigated, to which extent CRCs phenocopy normal colonic crypts. We categorize the degree of crypt-like architecture in a colorectal cancer case collection with long term follow-up data and demonstrate that *in vitro* and *in vivo* growth conditions strongly influence on this structural organization.

## Materials and Methods

### Histology and Immunohistochemistry

Formalin fixed, paraffin embedded normal human colonic mucosa, primary CRC tissues, xenograft tumors, and Matrigel embedded tumor cell spheroids were cut into 5 µm sections, deparaffinized, and antigens were retrieved in TRS6 (Dako Cytomation) for 20 min in a microwave oven. Adherent cultures of Caco2 and SW1222 cells were grown on cover slips, fixed in formalin, and permeabilized in 2% TritonX-100. Slides or cover slips then were incubated sequentially with mouse anti-β-Catenin (BD Transduction Laboratories; 1∶200), rabbit anti-KI67 (Cell Signaling; 1∶200) and goat anti-CK20 (Santa Cruz Biotechnology; 1∶50) for 1 h each at room temperature, washed with PBS, and then with FITC conjugated donkey anti-goat (Jackson ImmunoResearch; 1∶200), AlexaFluor 555 conjugated goat anti-mouse, and AlexaFluor 405 conjugated goat anti-rabbit (Invitrogen; 1∶500). For visualization of nuclear β-Catenin in normal colonic crypts, nuclei were counterstained with DAPI (Vector Laboratories). Confocal fluorescence images were taken on a LSM 700 laser scanning microscope using the ZEN software (Carl Zeiss). For conventional and double immunostaining, prediluted mouse anti-β-Catenin (Ventana Medical Systems) and/or mouse anti-CK20 (Progen; 1∶200) were used, and staining was performed on a Ventana Benchmark XT autostainer with ultraView Universal DAB and alkaline phosphatase detection kits (Ventana Medical Systems). Isotype-controls were included for all antigens.

### Cell culture and mouse xenografts

Caco2 cells were purchased from the American Type Culture Collection and SW1222 cells were obtained from the Ludwig Institute for Cancer Research (New York, USA). Adherent cell cultures were grown in Dulbecco's Modified Eagle's Medium (DMEM, Biochrom), supplemented with 10% fetal bovine serum, 1% glutamine, and 1x Penicillin-Streptomycin. Spheroid cultures were grown in StemPro hESC SFM medium in the presence of 20 ng/ml EGF and 10 ng/ml bFGF (Life Technologies) in ultra-low attachment flasks (Corning). Vital tissue of a sporadic primary colon adenocarcinoma was acquired through the Stiftung Human Tissue & Cell Research (HTCR, München, Germany), disaggregated into single cell suspensions using collagenase IV (Worthington Biomedical) and DNase I (Sigma-Aldrich), as previously described [10], and expanded in spheroid cultures. For further analyses, spheroids of cell lines and primary tumor cells were harvested by centrifugation at 200 g, resuspended in Matrigel, fixed in paraformaldehyde, and then sandwiched between HistoGel layers (ThermoScientific) for paraffin embedding. For xenograft growth, 10^6^ cells from adherent cell cultures or 10^5^ spheroid-derived primary colon cancer cells were suspended in 100 µl of a 1∶1 mixture of PBS and growth factor-depleted Matrigel (BD Bioscience) before subcutaneous injection into 6–8 week old NOD/SCID (NOD.CB17-Prkdc^scid^, The Jackson Laboratory) mice. Animals were housed in pathogen free micro-isolator cages and sacrificed when xenografts reached a diameter of 1 cm. Xenograft tumors then were removed, formalin fixed, and paraffin embedded. Three independent xenografts were subjected to further investigation for each cell line or primary tumor. Experimental procedures using animals were reviewed and approved by the Regierung von Oberbayern.

### CRC specimen collection and statistics

CRC specimens from patients that underwent intentionally curative surgical resection between 1994 and 2006 at the LMU were drawn from the archives of the institute of pathology. Follow-up data were recorded by the Munich Cancer Registry. Specimens and data were anonymized, and the need for consent was waived by the institutional ethics committee of the Medical Faculty of the LMU. Inclusion criteria were localized colorectal adenocarcinomas with absence of nodal (N0) or distant metastasis (M0) at the time of diagnosis (UICC stage I and II). Tumor tissues were assembled into tissue microarrays (TMAs) with 6 representative 1 mm cores, including tumor edges and tumor centers of each case. The final collection consisted of 221 CRC cases of which in 41 cases (19%) patients had died of their tumor within the follow-up period. Survival data were censored when case follow-up was discontinued or patients had died of reasons other than CRC. Case characteristics are summarized in Table 1. TMA slides then were subjected to single and double stainings for β-Catenin and CK20, as described above. Whole tissue sections of individual cases with heterogeneous β-Catenin and CK20 distribution were used for triple immune fluorescence analyses. Cancer specific survival was analyzed by the Kaplan-Meier method and groups were compared with the log-rank test. Statistics were calculated using SPSS (IBM).

**Table 1:** Clinicopathological variables and association with crypt-like type A morphology in colorectal cancer.

Characteristics	Total	Type A tumor		p
		yes	no	
All patients	221 (100)	75 (33.9)	146 (66.1)	
Age (y, median 69)				
≤68	110 (49.8)	40 (18.1)	70 (31.7)	0.45
≥69	111 (50.2)	35 (15.8)	76 (34.4)	
Gender				
Male	121 (54.8)	45 (20.4)	76 (34.4)	0.26
Female	100 (45.2)	30 (13.6)	70 (31.7)	
Tumor size (UICC)				
T1	1 (0.5)	0 (0)	1 (0.5)	0.61
T2	36 (16.3)	15 (6.8)	21 (9.5)	
T3	176 (79.6)	58 (26.2)	118 (53.4)	
T4	8 (3.6)	2 (0.9)	6 (2.7)	
Tumor grade (WHO)				
low grade	200 (90.5)	72 (32.6)	128 (57.9)	0.046
high grade	21 (9.5)	3 (1.4)	18 (8.1)	

Percent values are given in parentheses.

## Results

### Crypt-like functional compartments and axis formation in colorectal cancer

**Figure 1:**
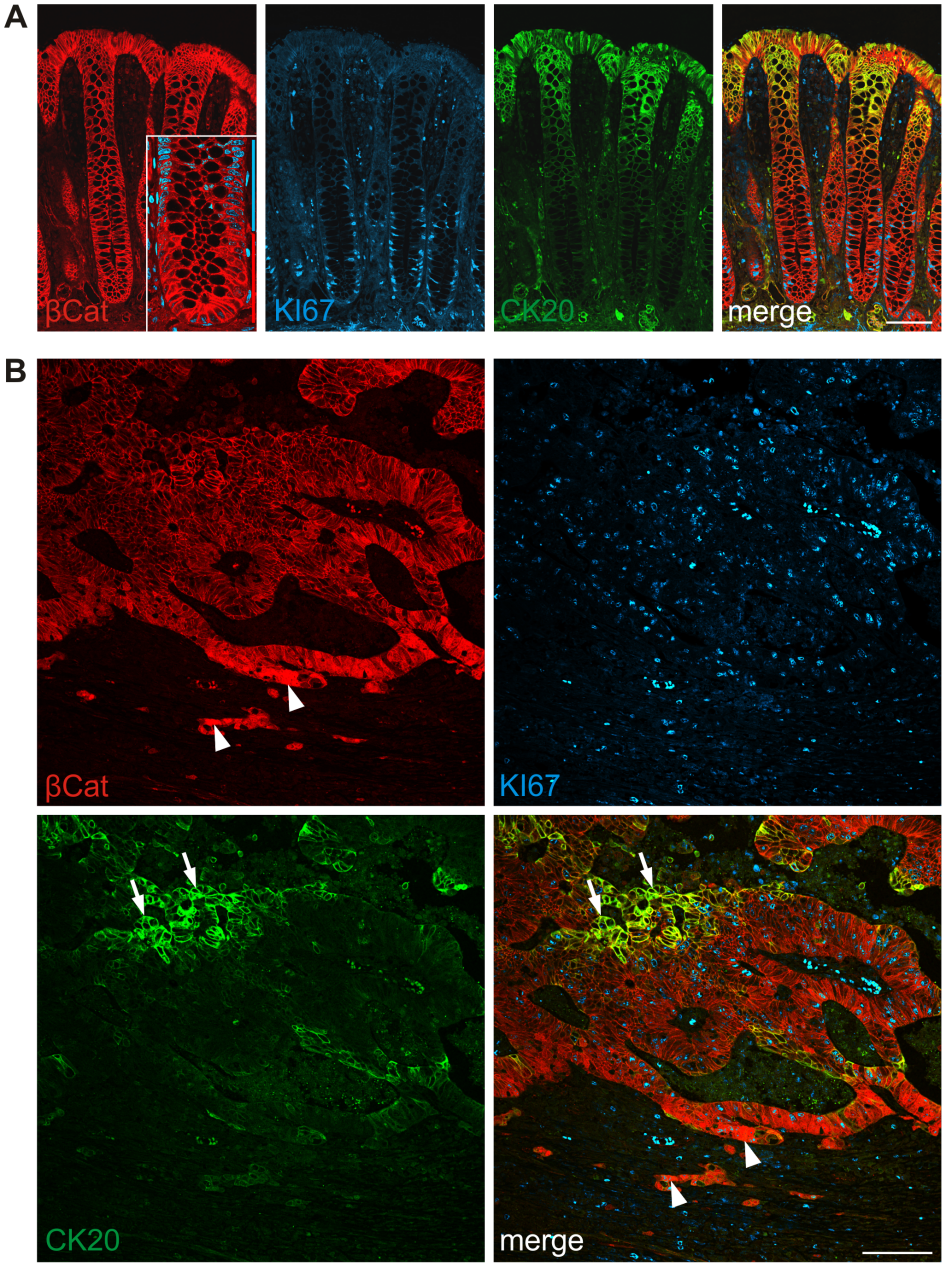
Colorectal cancers phenocopy normal colonic crypt architecture. Triple immune staining for β-Catenin (βCat), KI67 and CK20. (**A**) Colonic crypts show nuclear β-Catenin expression at their base, KI67 extending to the mid crypt, and CK20 at the apex. Visualization of nuclear β-Catenin required counterstaining with DAPI and high magnification (*left panel inset*). Red and blue bars delineate crypt parts with and without nuclear β-Catenin, respectively. (**B**) Colorectal cancers display a similar organization with nuclear β-Catenin at the tumor edge (*arrowheads*), CK20 expression in the tumor center (*arrows*), and KI67 in between. *Scale bars*, 100 µm.

To visualize different functional compartments within normal colonic crypts and CRCs, we established triple immune fluorescence stainings for β-Catenin, proliferation- related Ki-67 antigen (KI67) and Cytokeratin 20 (CK20). As expected, confocal imaging of normal colonic crypts indicated WNT active compartments with nuclear expression of β-Catenin at the crypt base, a KI67 expressing transit amplifying zone of proliferating epithelial progenitors, and most differentiated epithelial cells expressing CK20 towards the crypts' apices (Figure 1A). Notably, visualization of weak but distinct nuclear β-Catenin at the crypt base required high magnifications and slight nuclear counterstaining (Figure 1A, Figure S1). We then subjected primary CRCs to the same triple staining protocol. Similar to previous findings [8], [9], strong nuclear expression of β-Catenin was confined to tumor cells at the leading tumor edge and included morphologically undifferentiated colon cancer cells infiltrating surrounding stromal tissue (Figure 1B). Interestingly, and on the contrary to nuclear β-Catenin, CK20 staining was most intense in more central and glandular differentiated tumor epithelia, neighboring tumor necrosis (Figure 1B). KI67 was found between these zones and was reduced in tumor cells with strong CK20 expression (Figure 1B). These findings indicated that, despite disarrayed gland formation, colon cancers preserve some degree of structural organization and axis formation found in normal colonic crypts.

### Xenograft models of colon cancers reproduce the full spectrum of crypt-like organization

**Figure 2:**
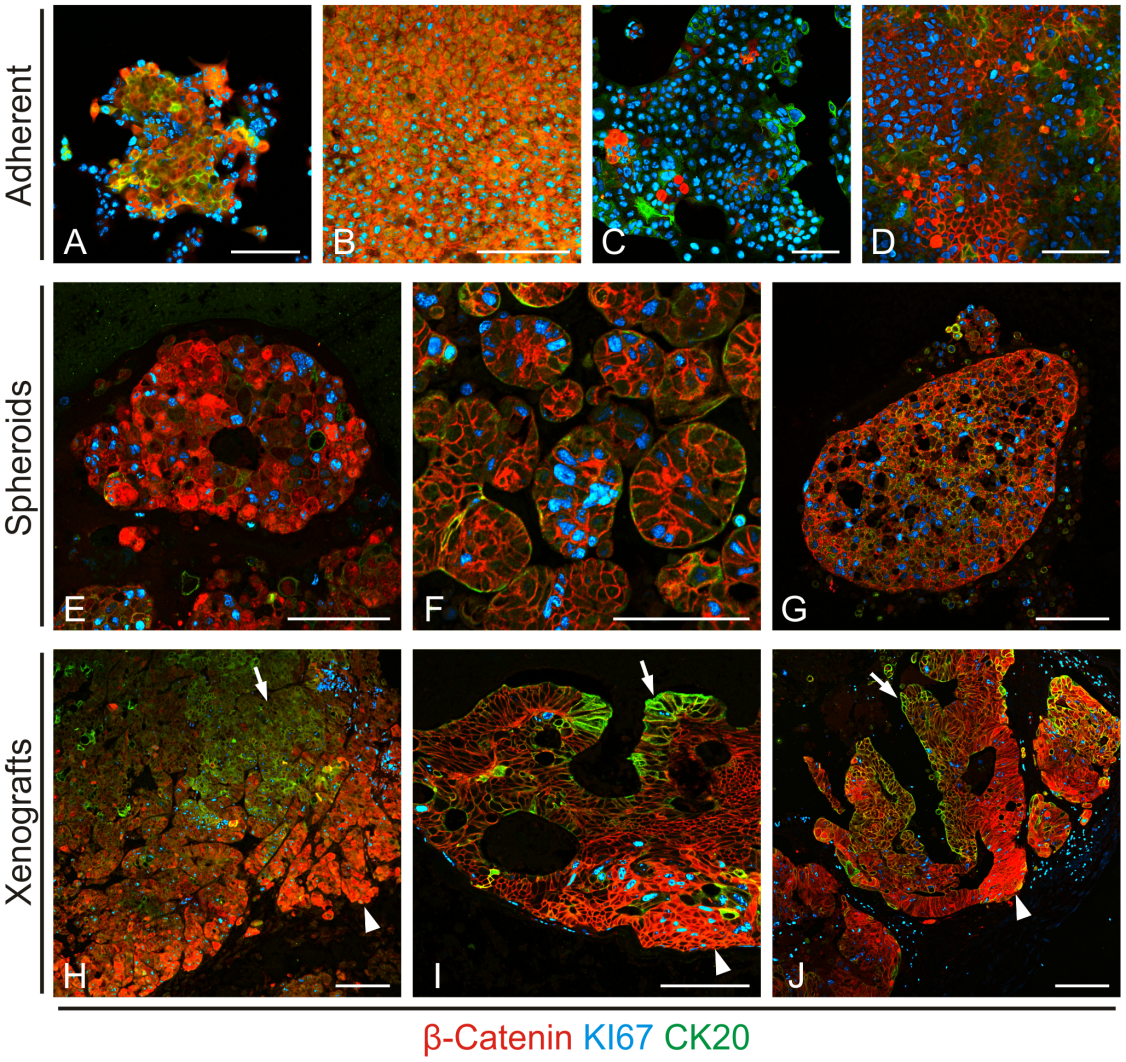
Impact of different culture conditions on colon cancer architecture. Triple immune staining reveals lack of full structural organization in adherent tissue cultures of (**A**) low density and (**B**) confluent SW1222, and (**C**) low density and (**D**) confluent Caco2 colon cancer cells, as well as in spheroid cultures of (**E**) SW1222, (**F**) Caco2, and (**G**) primary colon cancer cells. On the contrary, subcutaneous xenografts of (**H**) SW1222, (**I**) Caco2, and (**J**) primary colon cancer cells form glands with organized expression of nuclear β-Catenin at the tumor edge (*arrowheads*) and CK20 expression in the tumor center (*arrows*). *Scale bars*, 100 µm.

Next, we analyzed a primary colon cancer and colon cancer cell lines under different growth conditions *in vitro* and *in vivo*. Adherent *in vitro* cell cultures of SW1222 and Caco2 colon cancer cell lines revealed some degree of tumor cell heterogeneity for nuclear β-Catenin, KI67 and CK20 that was more prominent in low density than in confluent cultures (Figures 2A-D). Low density SW1222 cultures also exhibited some rudimentary centripetal axis formation with slightly enhanced CK20 expression and reduced KI67 proliferation within the colony centers, and stronger nuclear β-Catenin staining at the colony edge (Figure 2A). In Caco2 cells, differential expression of CK20 and nuclear β-Catenin appeared more randomly distributed (Figure 2C). To assess whether three dimensional growth would yield higher degrees of structural organization and tumor cell heterogeneity *in vitro*, we cultured both cell lines and primary colon cancer cells as non-adherent spheroids. Similar to our findings in adherent cultures, we observed some tumor cell heterogeneity for all three markers (Figures 2E-G). However, although primary colon cancer spheres showed slight centripetal axis formation with enhanced central CK20 staining (Figure 2G), no full structural organization, as we had found in primary colon cancer tissue, was observed. We then injected both cell lines and primary colon cancer cells into immune compromised mice for subcutaneous tumor formation *in vivo*. On the contrary to our *in vitro* findings, these tumors formed glandular structures with full degrees of tumor cell heterogeneity and distinct nuclear β-Catenin, KI67 and CK20 positive compartments (Figures 2H-J), mimicking those found in primary CRCs, and reminiscent of normal colonic crypt architecture. Collectively, these findings indicated that three dimensional growth and surrounding stromal tissue are required to expose full tumor cell heterogeneity and axis formation in CRCs.

### Classification and clinical relevance of crypt-like organization in colorectal cancers

**Figure 3:**
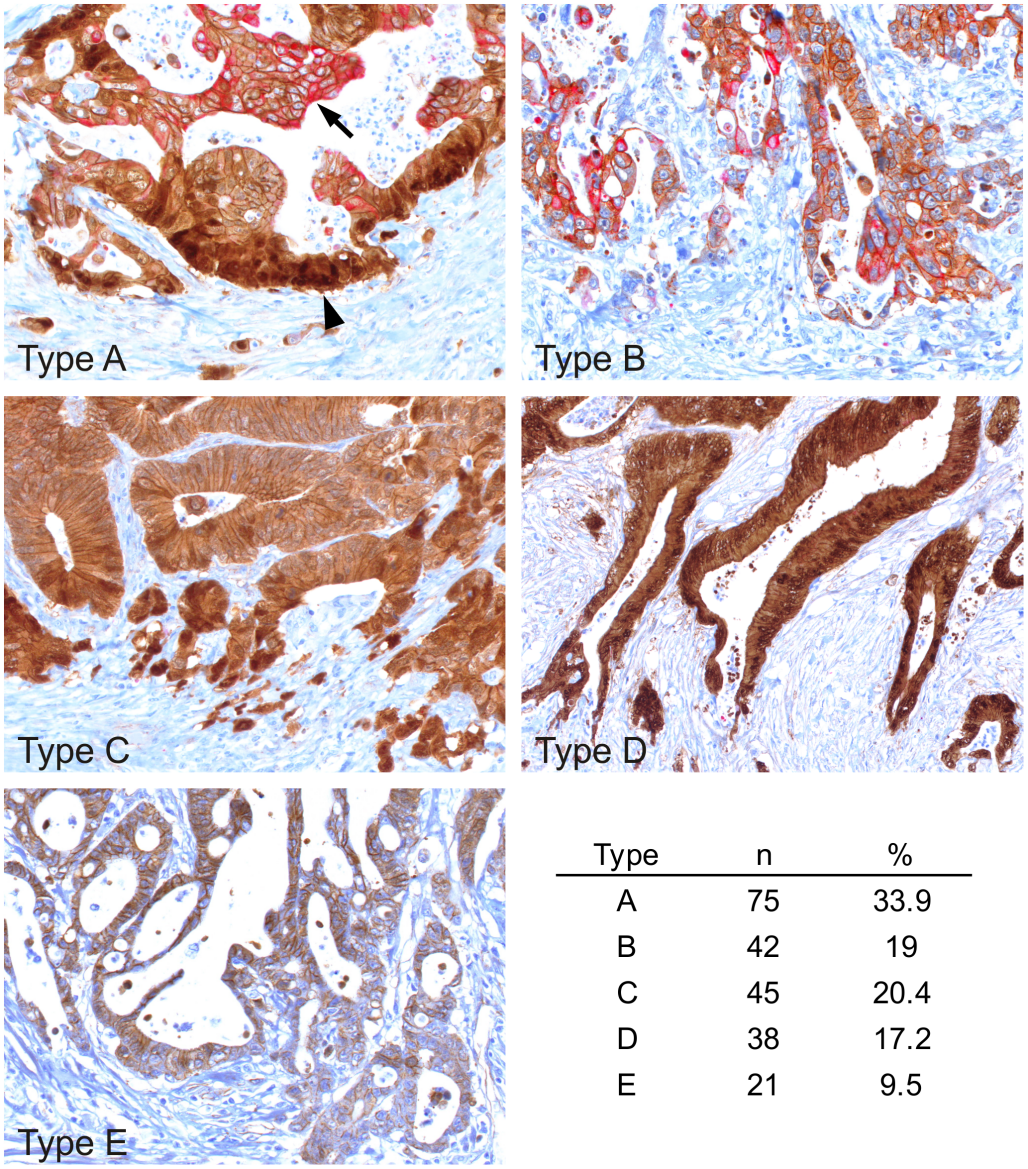
Types of colorectal cancer as defined by nuclear β-Catenin and CK20 expression. Double immune staining for β-Catenin (*brown*) and CK20 (*red*). (**Type A**) Full structural organization with nuclear β-Catenin at the tumor edge (*arrowhead*) and CK20 within the tumor center (*arrow*). (**Type B**) Absence of nuclear β-Catenin. (**Type C**) Absence of central CK20. (**Type D**) Absence of decreased nuclear β-Catenin in the tumor center. (**Type E**) Absence of both nuclear β-Catenin and CK20. Frequencies of these types are given in the table.

**Figure 4:**
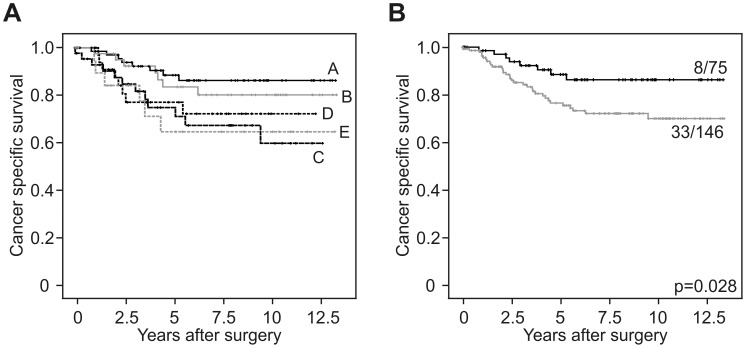
Type A colorectal cancers are associated with better survival. (**A**) Kaplan-Meier plot for the different types of colorectal cancers, as indicated by letters on curves. (**B**) Type A colorectal cancer patients (*upper curve*) show significantly (log-rank test) better survival when compared to other types (*lower curve*). Ratios on curves indicate the number of events over the number of patients per group.

To determine the frequency of crypt-like organization in CRCs, we used a case collection of 221 primary tumors and applied double immune staining for β-Catenin and CK20, marking both extremes of normal colonic crypt compartments. Analyses of these cases revealed 5 different types of colon cancers: The largest group (type A) showed organized expression of nuclear β-Catenin at the leading tumor edge and enhanced CK20 within the tumor center (Figure 3). Of note however, when examining cases of this type on serial sections, we observed additional enhanced CK20 expression in most infiltrative tumor cells at the leading tumor edge, overlapping with nuclear β-Catenin in most of these cases (Figure S2). The other tumors lacked this organization to some degree (Figure 3), either by absence of nuclear β-Catenin (type B), absence of enhanced CK20 in the tumor center (type C), absence of decreased nuclear β-Catenin in the tumor center (type D), or absence of both nuclear β-Catenin and CK20 expression (type E). We then examined these types for associations with tumor specific survival. Interestingly, Kaplan-Meier plots revealed best outcome for CRCs with full crypt-like structural organization, based on nuclear β-Catenin and CK20 expression (type A) that was significantly better, when statistically tested against all other types combined (Figure 4). We then examined type A tumors for associations with other clinical variables and found a significant correlation with low tumor grade, while there was no significant association with other variables such as age, gender and T category (Table 1). Finally, when testing type A tumors against others for survival prediction in multivariate analyses, full structural organization proved to be an independent prognostic marker for better cancer specific survival (Table 2). These findings demonstrate that CRCs mimic crypt-like compartments and axis formation to varying degrees and implicate that high structural similarity to normal colonic crypts may be associated with less aggressive tumor behavior.

**Table 2:** Multivariate analysis of cancer specific survival.

Variables	Cancer specific survival		
	HR	(95% confidence interval)	p
Age (y)	2.0	(1.02–3.81)	0.045
Gender	1.2	(0.63–2.36)	0.55
T category	3.5	(1.40–8.55)	0.007
Tumor grade	1.3	(0.53–2.97)	0.6
Type A tumor	0.5	(0.21–0.998)	0.049

## Discussion

Here, we demonstrate that CRCs phenocopy normal colonic crypt architecture. In contrast to normal crypts, these tumors grow invasively and form morphologically disarrayed glands [7]. However, distinct compartments with high WNT activity at the leading tumor edge, adjacent proliferation, and enhanced CK20 expression towards the tumor center can be identified. Since normal colonic crypts have a known WNT driven stem cell compartment at their base, maturing into epithelial cells with strong CK20 expression at the apex [4]–[6], our findings support the idea of colon cancers mimicking these functional compartments, with colon cancer stem cells at the leading tumor edge, and centripetal tumor cell differentiation, as was previously hypothesized [13]. However, our data also indicate significant discrepancies to normal colonic crypt architecture, since not all CRCs showed the full range of crypt-like compartmentalization. Moreover, CRCs with discernable compartments frequently showed enhanced CK20 not only in central tumor areas but additionally at the leading tumor edge, coinciding with strong nuclear β-Catenin and high WNT activity. While these findings implicate that morphogenetic programs of intestinal epithelial cells are still active in colorectal cancers to some extent, markers for terminal differentiation in the normal intestine may not necessarily be restricted to fully differentiated tumor cells of intestinal neoplasms.

Comparing different culture and growth conditions of colon cancer cells, we observed fully developed and compartmentalized glandular structures in *in vivo* xenograft tumors only. Recapitulation of structures, imitating normal colonic crypt architecture and full gradient formation of least and most differentiated colon cancer cells, may therefore require signaling from a tumor microenvironment [8], [14], missing in standard culture systems. These findings have important implications for studies that focus on specific tumor cell subpopulations, since tumor cell composition in regular or spheroid cultures may not adequately reflect the situation in primary tumors. Interestingly however, when grown as xenograft tumors, even established colon cancer cell lines formed fully developed crypt-like structures and compartmentalization, similar to primary colon cancers. This suggests that, despite some expected genetic alterations in passaged cell lines [15], using colon cancer cell line xenografts may not be inferior to the use of primary colon cancers for studies on tumor cell subpopulations, such as colon cancer stem cells [12], [16]. In this context, growth conditions *in vivo* may be more relevant than the source of the colon cancer cell, either derived from established cultures or primary tumors.

When looking at associations with clinical parameters, we linked crypt-like structural organization of CRCs to improved survival and low tumor grade. While prognosis of these tumors can best be estimated by staging, describing extent of the disease [17], histopathological tumor grading has independently been linked with disease outcome and reflects the overall degree of tumor cell differentiation [18]. Several grading systems based on different aspects of tumor morphology have been proposed, however, due to poor reproducibility, a simplified two-tiered system, only considering gland formation, is currently favored, and classifies these tumors into low and high grade neoplasms [19]. Although this grading system is widely accepted and regarded as a consistent prognostic factor by the College of American Pathologists, it does not integrate existence and distribution of distinct tumor cell subpopulations that may impact on prognosis. On the other hand, recently emerging approaches to assess CRC prognosis, such as stem cell marker expression [20], [21] or microRNA profiling [22], so far either lack general acceptance or have the caveat of being costly and technically demanding. Since in our study, crypt-like organization served well in predicting disease outcome, we propose that this approach may integrate morphological aspects of gland formation with markers indicating functional tumor compartments. β-Catenin and CK20 expression can be robustly assessed in colon cancer specimens and integration of these parameters into routine diagnostics may therefore improve histopathological colon cancer grading. In addition to these considerations, our findings may also explain previous inconsistent data on clinical correlates of nuclear β-Catenin [23], [24], tumor cell proliferation [25], [26], and CK20 expression in colon cancer [27], [28]. If these markers indicate functional and structural compartments within these tumors, it is possible that compartment organization is more relevant to tumor biology and reflected in clinical outcome than simple compartment size.

## Supporting Information

Figure S1 **Increased WNT-activity at the base of normal colonic crypts.** While β-Catenin labels epithelial cell membranes as part of adherens junctions, high magnifications indicate additional nuclear β-Catenin expression at the crypt base (*black arrowheads*). Lack of nuclear expression with dominance of blue counterstaining is seen in epithelial cells above the crypt base (*blue arrowheads*). *Left panel* shows higher magnification of area boxed in *right panel*.
(TIF)

Figure S2 **CK20 expression is not limited to the tumor center of type A colorectal cancers.** Serial sections demonstrate, in addition to enhanced CK20 staining in the tumor center (*arrows*), enhanced staining for CK20 at the tumor edge, overlapping with nuclear β-Catenin (*arrowheads*). This pattern was found in 61% of type A colorectal cancers.
(TIF)
