# Supplementary figures and images for: Colorectal Cancers Mimic Structural Organization of Normal Colonic Crypts
Source: PLoS One. 2014 Aug 11;9(8):e104284. doi: 10.1371/journal.pone.0104284 (PMC4128715; doi:10.1371/journal.pone.0104284)

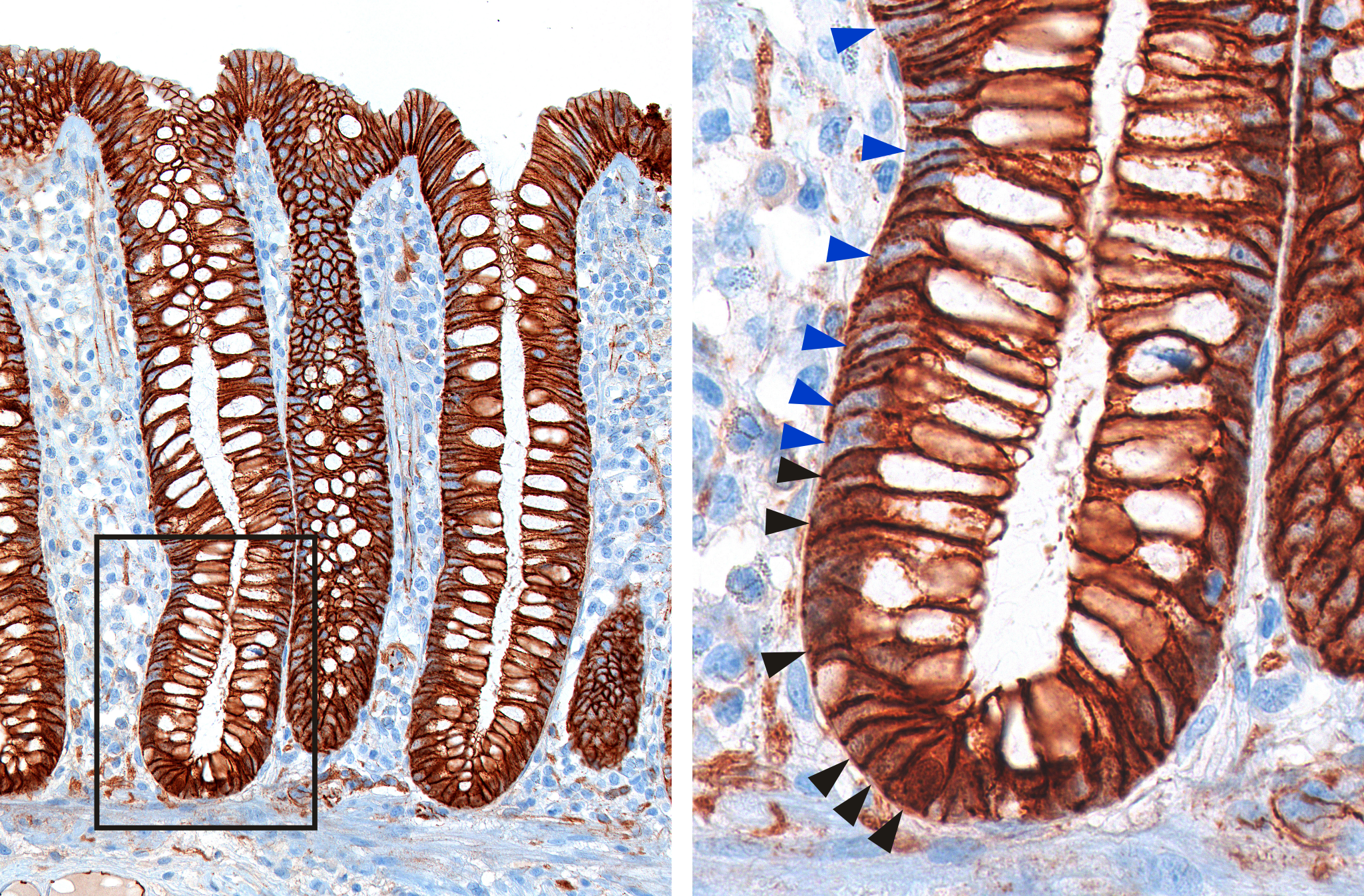

Supplement: Figure S1 — Increased WNT-activity at the base of normal colonic crypts. While β-Catenin labels epithelial cell membranes as part of adherens junctions, high magnifications indicate additional nuclear β-Catenin expression at the crypt base (black arrowheads). Lack of nuclear expression with dominance of blue counterstaining is seen in epithelial cells above the crypt base (blue arrowheads). Left panel shows higher magnification of area boxed in right panel. (TIF) [file pone.0104284.s001.tif]

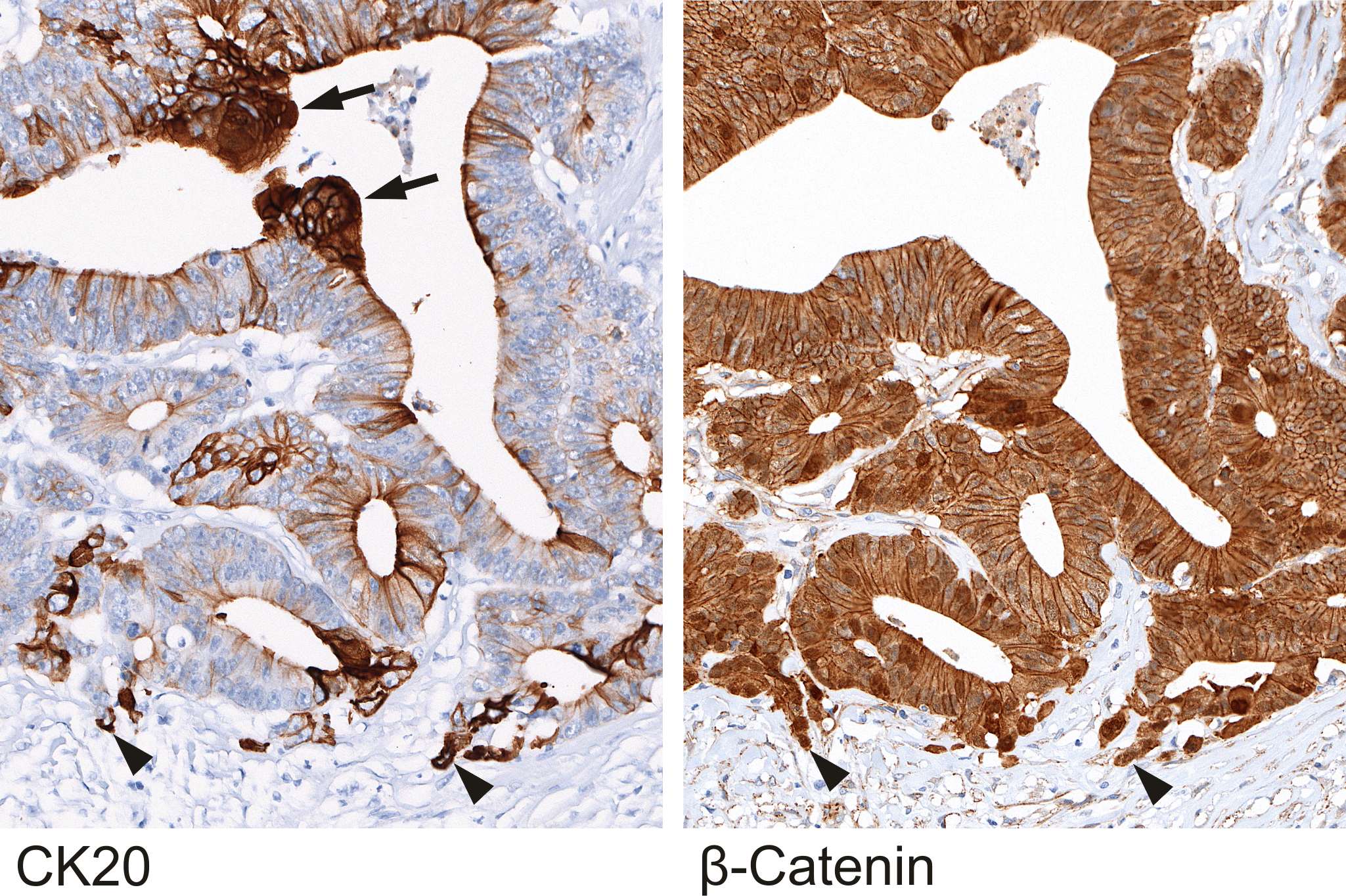

Supplement: Figure S2 — CK20 expression is not limited to the tumor center of type A colorectal cancers. Serial sections demonstrate, in addition to enhanced CK20 staining in the tumor center (arrows), enhanced staining for CK20 at the tumor edge, overlapping with nuclear β-Catenin (arrowheads). This pattern was found in 61% of type A colorectal cancers. (TIF) [file pone.0104284.s002.tif]
